# Evaluation of Two Rapid Lateral Flow Tests and Two Surrogate ELISAs for the Detection of SARS-CoV-2 Specific Neutralizing Antibodies

**DOI:** 10.3389/fmed.2022.820151

**Published:** 2022-02-04

**Authors:** Philipp Girl, Katrin Zwirglmaier, Heiner von Buttlar, Roman Wölfel, Katharina Müller

**Affiliations:** ^1^Department of Virology and intracellular Pathogens, Bundeswehr Institute of Microbiology, Munich, Germany; ^2^Department of Bacteriology and Toxinology, Bundeswehr Institute of Microbiology, Munich, Germany; ^3^Department of Diagnostics, Innovation and Verification, Bundeswehr Institute of Microbiology, Munich, Germany; ^4^German Centre for Infection Research (DZIF), Partner Site Munich, Munich, Germany

**Keywords:** COVID-19, SARS-CoV-2, neutralizing antibodies, ELISA, lateral flow assay

## Abstract

As vaccination against SARS-CoV-2 progresses rapidly around the world, reliable detection of SARS-CoV-2 specific neutralizing antibodies (NAb) has become an indispensable component of serological diagnostics. We evaluated the performance of four commercially available tests, i.e. two lateral flow assays (Coris BioConcept COVID-19 Sero NP/RBD and Concile InfectCheck COVID-19 NAb) and two surrogate ELISA (sELISA) tests (EUROIMMUN SARS-CoV-2 NeutraLISA and AdipoGen SARS-CoV-2 Neutralizing Antibodies Detection Kit) in comparison with an in-house SARS-CoV-2 micro neutralization test as reference. A total of 334 sera were tested, including 30 samples collected prior to the emergence of SARS-CoV-2, 128 sera from convalescent patients as well as 176 sera from partially or fully vaccinated individuals. The overall sensitivity of LFAs differed and was 71.6% for the Coris and 98.4% for the Concile. In contrast, overall sensitivity of the NeutraLISA was 86 and 98% for the AdipoGen. All test showed the highest sensitivity when testing samples from fully vaccinated individuals with both sELISA achieving 100% sensitivity. Overall specificity was 89.3% for the Coris and only 58.3% for the Concile. Similarly, significant differences were observed for both sELISA, with an overall specificity of 82.1% for the NeutraLISA and only 54.8% for the AdipoGen. All tests showed a 100% specificity when testing negative control samples while specificities were lowest when testing samples from only partially vaccinated individuals.

## Introduction

Serological testing has become a useful tool in the fight against the SARS-CoV-2 pandemic. Among others, it can be used to estimate the prevalence and incidence in a given population (1, 2). Countless serological tests have been commercialized by now, including enzyme-linked immunosorbent assays (ELISAs) as well as point-of-care lateral flow assays (LFAs). These tests typically measure SARS-CoV-2 specific antibodies, often even specific immunoglobulin subclasses (i.e. IgG, IgM or IgA). In this context, neutralizing antibodies (NAbs) have emerged as a strong correlate of protection and are therefore being used to evaluate vaccine efficacy or select appropriate convalescent plasma for therapeutic use (3). However, they represent only a small subset of the total polyclonal immune response.

The main target of NAbs is the spike (S) protein, more precisely the Receptor Binding Domain (RBD) located at the outer end of the S1 subunit. This is responsible for the binding of SARS-CoV-2 to the Angiotensin-Converting Enzyme-2 (ACE2) receptor on the surface of the cell (4). Thus, it is not surprising that a recent study found that at least 90% of SARS-CoV-2 NAbs are RBD specific. However, not all RBD-specific antibodies are able to neutralize the virus (5). Thus, to efficiently assess the level of protection from (re-)infection rather than just demonstrate a prior encounter with SARS-CoV-2, serological tests need to be able to differentiate between neutralizing and non-neutralizing antibodies.

Plaque-reduction (PRNT) and neutralization (NT) tests are the current gold standards for the detection and quantification of NAbs (6). However, these assays are labor-intensive and require appropriate biocontainment laboratories (BSL-3) because they depend on working with infectious virus. The number of laboratories that can perform these tests is therefore limited (7). Previously described pseudo virus-based techniques can be performed under BSL-2 conditions, but also require the cultivation of reporter virus and therefore do not provide a significant time advantage (8, 9). A potential alternative to classic neutralization assays comes in the form of surrogate ELISA (sELISA). They rely on the ability of NAbs to bind RBD and prevent its interaction with ACE2. sELISAs use recombinant proteins and colorimetric analysis to determine this competitive inhibition and several versions of this type of assay have been commercialized (10). In addition, LFAs have been developed and are particularly appealing as point-of-care (POC) tests due to their rapid turnaround times and simplicity of use (11). In contrast to sELISAs, they are designed as sandwich assays to detect RBD-specific antibodies with neutralizing capability.

In this study, we investigated four commercially available tests for the detection of SARS-CoV-2 specific NAbs: two surrogate sELISAs (Euroimmun SARS-CoV-2 NeutraLISA and AdipoGen SARS-CoV-2 Neutralizing Antibodies Detection Kit) and two LFAs (Coris BioConcept COVID-19 Sero NP/RBD and Concile InfectCheck COVID-19 NAb). According to the manufacturers, all four tests are suitable for the detection of antibodies with neutralizing capabilities. However, the conceptual design differs between both types of tests. Both sELISAs are very similar in design and imitate the interaction of RBD and ACE2 to determine the inhibitory effect of neutralizing antibodies capable of interrupting this interaction. Based on the information provided by the manufacturers both LFAs simply detect RBD-specific antibodies without assessing functionality.

We tested all four assays side-by-side and in direct comparison with our in-house NT to determine both sensitivity and specificity and to test their potential as an alternative to conventional NT. While several studies have attempted to compare SARS-CoV-2 immunoassays in the past (12–14), our study focuses on immunoassays designed to detect antibodies with neutralizing function rather than antibodies in general. Furthermore, our study population differs from previous studies in that we specifically included samples from vaccinated individuals in addition to convalescent individuals in order to also investigate the potential use of such assays in the context of vaccine evaluation as well as the evaluation of the humoral immune response post vaccination.

## Materials and Methods

### Serum Samples

**Figure 1:**
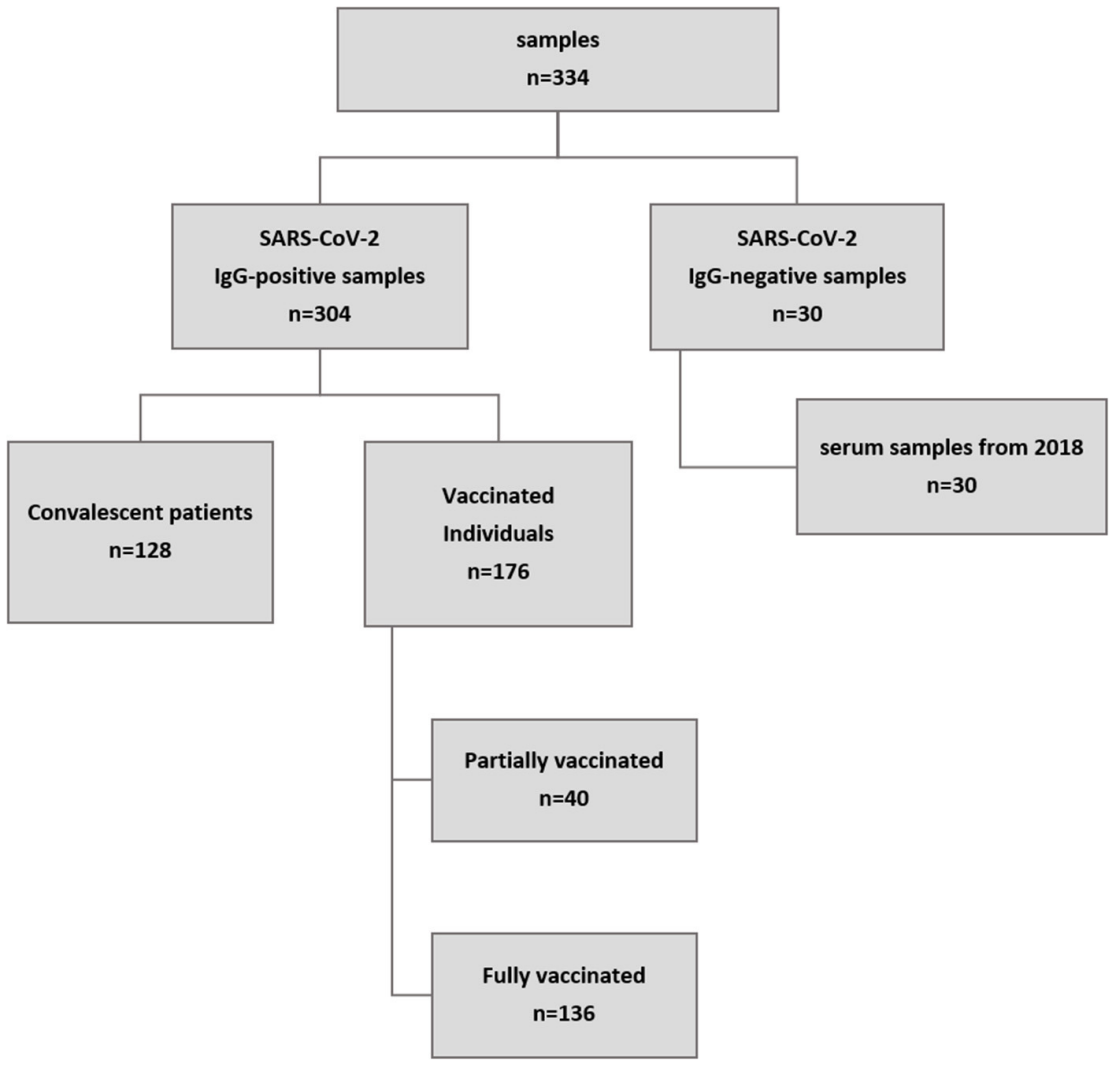
Overview of sample origin. A total of 334 samples were tested. A commercial IgG ELISA (Euroimmun, Lübeck, Germany) was used for pre-screening. All but 30 samples were positive for SARS-CoV-2 IgG antibodies, of which 128 came from patients who had recently recovered from confirmed COVID-19 and 177 were from vaccinated individuals (collected at least three weeks after vaccination). In addition, 30 samples collected in mid to late 2018 served as negative control samples.

We tested a total of 334 human serum samples. Samples were selected by non-probability sampling (convenience/purposive sampling). Of these samples, 304 had previously tested positive for SARS-CoV-2 antibodies in a commercial IgG ELISA (Euroimmun, Lübeck, Germany), which was used as a pre-screening assay (Supplementary Table 1). Of these, 128 samples came from PCR-confirmed convalescent patients of which 78 were registered convalescent plasma donors; all samples were taken six weeks after full recovery according to the official guidelines provided by the German Federal Institute for Vaccines and Biomedicine (15). All samples were from patients, which were hospitalized for their SARS-CoV-2 infection, but did not require mechanical ventilation. Another 177 samples came from vaccinated individuals and were taken between four and six weeks after vaccination. Of these, 40 individuals had received their primary vaccination with Vaxzevria (AstraZeneca) while the other 136 individuals were fully vaccinated either with Comirnaty (Pfizer/BioNTech), Spikevax (Moderna) or a heterologous vaccination with Vaxzevria (AstraZeneca) as prime and Spikevax as boost. The remaining 30 serum samples were collected before the occurrence of SARS-CoV-2 (mid to late 2018) and served as negative control samples. An overview of all samples is given in Figure 1.

All samples evaluated were residual diagnostic material. Therefore, no specific information (e.g. demographic characteristics, individual disease progression etc.) could be assigned to individual samples or patients.

### Ethical Statement

The study was carried out in-line with “The Code of Ethics of the World Medical Association (Declaration of Helsinki)”. The use of serum samples complied with the guidelines of the Central Ethics Committee of the German Medical Association [Dtsch Arztebl 2003; 100(23): A-1632]. In accordance with these guidelines, the anonymized use of residual material from the samples sent to our laboratory for diagnostic purposes is permissible, provided that the patients have not decided against this procedure. Samples from patients who had decided against this procedure were excluded from the analyses.

### Neutralization Test (NT)

SARS-CoV-2 NAb titers were determined as previously described (16). Briefly, serum samples (duplicates) were serially diluted in 96-well tissue culture plates starting at 5 to a maximum of 640 along with positive and negative control samples. SARS-CoV-2 stocks (50 TCID/50 μl, MUC-IMB-1 [EPI_ISL_406862]) were prepared on Vero E6 cells and aliquots were stored at −80°C until further use. Virus was pre-incubated (1 h, 37°C) with diluted serum samples before Vero E6 cells (1 x 10e4 cells/50 μl) were added to each well. After 72 h (37°C), supernatants were discarded and wells were fixed (13% formalin/PBS) and stained with crystal violet (0.1%). The NAb titer corresponded to the reciprocal of the highest sample dilution showing complete inhibition of cytopathic effects. A virus re-titration was performed in triplicates on every plate and exact titers were determined by retrograde calculation.

### Surrogate ELISA (sELISA)

**Figure 2:**
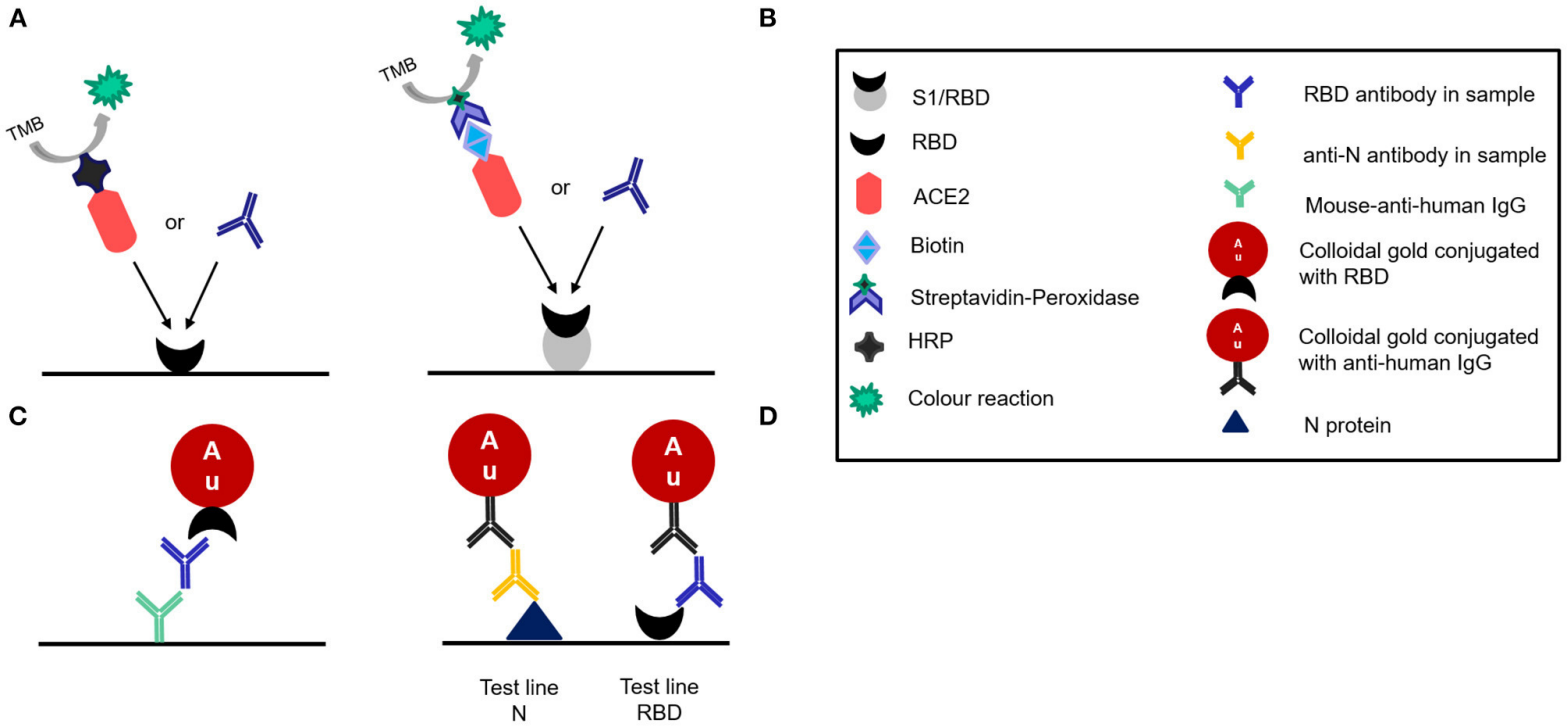
Schematic illustration of the different designs of the four tests evaluated as disclosed by the manufacturers. **(A)** Adipogen:Competitive ELISA **(B)** Euroimmun. NeutraLISA:competitive ELISA **(C)** Affimedix TestNow:Lateral flow rapid test **(D)** Coris Bioconcept:Lateral flow rapid test.

Two commercially available sELISA kits were tested in this study: The SARS-CoV-2 NeutraLISA (Euroimmun, Lübeck, Germany) and the SARS-CoV-2 Neutralizing Antibodies Detection Kit (AdipoGen Life Sciences, Liestal, Switzerland). Both tests function very similarly and are based on the competitive inhibition of the RBD-ACE2 interaction by NAbs. AdipoGen provides RBD-coated plates and uses HRP-conjugated ACE2 while Euroimmun uses the total S1 subunit of the S protein for coating and biotin-conjugated ACE2 (Figures 2A,B). Both tests were carried out strictly according to the manufacturer's instructions.

The NeutraLISA is the only test studied here that classifies results not only as positive or negative, but also as “indeterminate.” We followed a conservative approach for the evaluation of the test results and evaluated indeterminate results as negative.

The sensitivity and specificity of the SARS-CoV-2 NeutraLISA were specified by the manufacturer as follows: Sensitivity = 95.9%; Specificity = 99.7%.

For the SARS-CoV-2 Neutralizing Antibodies Detection Kit (AdipoGen) no data on sensitivity and specificity was provided by the manufacturer.

### Lateral Flow Assay (LFA)

Two commercially available LFAs were evaluated in this study: COVID-19 Sero NP/RBD test (Coris BioConcept, Belgium) and the InfectCheck COVID-19 NAb test (Concile GmbH Germany/Affimedix Inc. USA). Both are sandwich immuno assays. The Concile LFA uses colloidal gold particles that are conjugated with the RBD to bind to the NAb and mouse-anti-human IgG immobilized on the test line of the membrane to capture the NAb bound to the gold (Figure 2C). The Coris BioConcept detects not only RBD antibodies, but also antibodies against the nucleocapsid protein. Since all vaccines currently in use elicit an immune response against the spike protein, while an infection elicits an immune response against all components of SARS-CoV-2, the LFA can differentiate between immune response acquired through vaccination or infection. The test uses N protein and RBD immobilized on separate test lines to capture the respective antibodies (Figure 2D). The other half of the immune sandwich consists of gold particles that are presumably (not detailed in the instructions for use) conjugated with anti-human IgG.

Both tests were carried out according to manufacturer's instructions. Briefly, for the COVID-19 Sero NP/RBD test 30 μl serum were applied to the test cassette followed by 4 drops of buffer 10 s later. Results were read out after 15 min and photographically documented within 15–20 min. Line intensities were evaluated visually by comparison to a visual analog color scale (Supplementary Figure 1). Intensities on the scale range from 0–9 and were recorded separately for test line and control line. Values of 2 or greater were scored as positive. A value of 1 corresponds to a very faint line that could not be reliably identified by different test operators and was therefore scored as negative. For the InfectCheck COVID-19 Nab test 5 μl serum were mixed with 5 drops of buffer and applied to the test cassette. Results were read out visually after 15 min as described above and photographically documented within 15–20 min. In addition to that, results were also measured with the concile α1 reader.

The sensitivity and specificity of the COVID-19 Sero NP/RBD (Coris) were specified by the manufacturer as follows: Sensitivity NP = 95.2%, Sensitivity RBD = 91.9%; Specificity NP= 98.5% Specificity RBD = 100.0%.

The sensitivity and specificity of the InfectCheck COVID-19 NAb test (Concile) were specified by the manufacturer as follows: Sensitivity = 100.0%; Specificity = 98.67%.

### Statistical Analysis

All statistical analyses were performed using GraphPad Prism 8 for Windows 64-bit [Version 8.4.3 (686)]. Sensitivity was defined as the ability of a test to identify the presence of SARS-CoV-2 specific NAb as determined by NT. Specificity was defined as the ability of a test to identify the absence of SARS-CoV-2 specific NAb as determined by NT.

## Results

### Detection of SARS-CoV-2 Specific Neutralizing Antibodies by NT

Initially, all 334 samples were examined in our in-house NT. Serum samples with a titer ≥5 were considered positive as previously determined and described (17). In total, 84 samples tested negative for neutralizing antibodies while the remaining 250 tested positive. Among the samples that tested negative were all 30 negative control samples, 14 samples from convalescent patients and all serum sample from individuals who had only received their primary vaccination. Positive samples consisted of 114 sera from convalescent patients and 136 sera from fully vaccinated individuals. In summary, 100% of sera from fully vaccinated individuals and 89% of convalescent sera tested positive for NAbs while 100% of both negative control samples and samples from individuals that received their primary vaccination tested negative. An overview of the distribution of NT results by sample origin is given in Table 1.

**Table 1:** Distribution of the results (positive/negative) from the in-house NT in relation to sample origin.

**Sample origin**	**Number of samples**	**NT positive**	**NT negative**
convalescent patients	128	114	14
partially vaccinated individuals	40	0	40
fully vaccinated individuals	136	136	0
negative control samples	30	0	30
Total	334	250	84

*All (100%) of the NT positive samples came from fully vaccinated individuals and convalescent patients while the majority (83.3%) of NT negative samples came from only partially vaccinated individuals and negative control samples*.

### Influence of Natural Infection and Immunization on Neutralizing Antibody Titers

**Figure 3:**
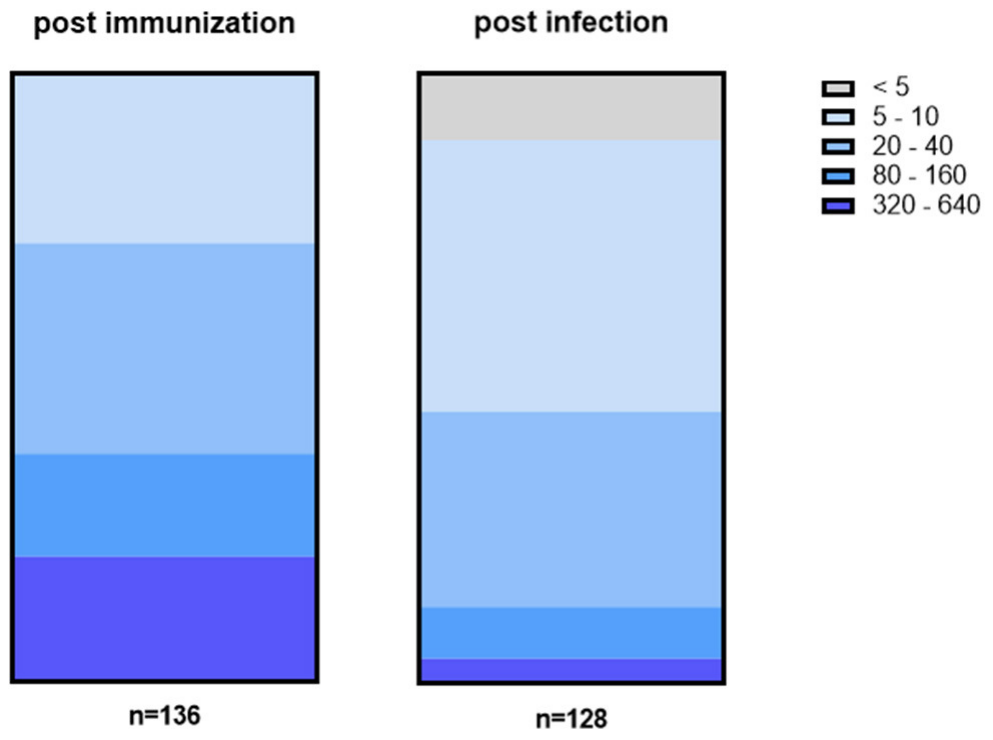
Titer values among fully vaccinated (left) differ from titer values of recovered patients (right). In general, titers were higher among fully vaccinated individuals with 38% having a titer of at least 80 or higher including 21% with a titer of ≥320. In contrast, 45% of convalescent patients had a titer of 5 or 10 while in 11% no NAbs could be detected at all.

The titer values determined by NT significantly differ between convalescent patients and fully vaccinated individuals. While the median titer of sera from convalescent patients was 37, it was 3.5 times higher (129) for sera from vaccinated individuals. Figure 3 shows the distribution of titer values among fully vaccinated individuals (left) and recovered patients (right).

Among serum samples from vaccinated individuals, one-third (28%) have low titers of 5 to 10. At the same time, 34% show a slightly higher titer of either 20 or 40 while the remaining samples (38%) have a titer ≥80. In more detail, 17% of vaccinated individuals have a titer between 80 and 160 while another 21% reached the highest titer values of 320 or more. In contrast, only 4% of convalescent patients have titer values that high while another 8% have a titer between 80 and 160. Instead, the majority (45%) of convalescent patients have a titer of either 5 or 10 and another one third (32%) have a titer of 20 to 40. In addition, no NAbs at all could be detected in 11% of sera.

### Sensitivity and Specificity of Commercial Immunoassays

In addition, all 334 samples were subsequently also analyzed using two sELISAs (AdipoGen and NeutraLISA) as well as two LFAs (Coris and Concile). Sensitivity and specificity were determined in direct comparison to the results of the NT. A detailed overview of the results of the individual immunoassays, including subgroup specific results, is shown in Table 2. In addition, a detailed overview of the distribution of inhibition values of both sELISAs within NT positive and NT negative samples as well as a two dimensional distribution of inhibition values is given in Supplementary Figure 2.

**Table 2:** Detailed overview of overall sensitivity and specificity of the four evaluated commercial immunoassays in comparison to NT results.

			**sELISA**		**LFA**	
**NT**			**AdipoGen**	**NeutraLISA**	**Coris**	**Concile**
Sensitivity: positive samples (titer ≥ 5)	all samples	*n = 250*	98% (245/250)	86% (215/250)	71.6% (179/250)	98.4% (246/250)
	convalescent	*n = 114*	95.6% (109/114)	69.3% (79/114)	65.8% (75/114)	97.4% (111/114)
	fully vaccinated	*n = 136*	100% (136/136)	100% (136/136)	76.5% (104/136)	99.3% (135/136)
Specificity: negative samples (titer <5)	all samples	*n = 84*	54.8% (46/84)	82.1% (69/84)	89.3% (75/84)	58.3% (49/84)
	convalescent	*n = 14*	42.9% (6/14)	100% (14/14)	85.7% (12/14)	42.9% (6/14)
	partially vaccinated	*n = 40*	25% (10/40)	62.5% (25/40)	82.5% (33/40)	32.5% (13/40)
	control samples	*n = 30*	100% (30/30)	100% (30/30)	100% (30/30)	100% (30/30)

With regard to the entirety of samples examined all but five NT-positive samples were identified by the AdipoGen sELISA, resulting in the overall highest sensitivity of 98%. In comparison, of the 250 NT-positive samples, 215 samples tested positive in the NeutraLISA giving an overall sensitivity of 86%. However, of the 84 NT-negative samples, 38 tested positive in the AdipoGen whereas the NeutraLISA detected NAbs in 15 of them, giving an overall specificity of 54.8% (AdipoGen) and 82% (NeutraLISA) respectively.

Among vaccinated individuals both sELISAs detected NAbs in all 136 NT-positive samples resulting in a sensitivity and specificity of 100%. At the same time, of the 114 NT-positive samples from convalescent patients, the AdipoGen identified 109 samples as positive (95.6% sensitivity) while the NeutraLISA detected 79 positive samples (69.3% sensitivity). In addition, of the 14 NT-negative samples from convalescent patients the AdipoGen identified more than half (*n* = 8) as positive (42.9% specificity) while NeutraLISA detected no NAbs in all 14 samples (100% specificity). Of the 30 negative control samples, all were identified as negative by both sELISAs resulting in 100% specificity each.

With regard to both LFAs tested, sensitivity and specificity also varied between tests. The Concile identified all but four NT positive samples resulting in the highest overall sensitivity (98.4%) of all four tests evaluated. At the same time, the Coris identified significantly less NT positive samples, resulting in an overall sensitivity of 71.6%. Similar results were obtained among samples from convalescent and fully vaccinated individuals with the Concile showing continuously high sensitivities (97.4–99.3%). In contrast, the Coris showed the lowest sensitivities among all four tests (65.8–76.5%). Overall, all tests achieved their highest sensitivity with samples from fully vaccinated individuals while sensitivities were lowest when testing samples from convalescent patients. In terms of specificity, the Concile identified 35 of 84 negative samples as positive resulting in an overall specificity of 58.3%. Similar results were obtained among samples from convalescent (42.9%) and partially vaccinated (32.5%) individuals. In contrast, a specificity of 100% was achieved when testing negative control samples. At the same time, of the 84 negative samples the Coris identified all but nine samples as negative, giving an overall specificity of 89.3%. Similar results were obtained for samples from convalescent patients (85.7%), partially vaccinated individuals (82.5%) and control samples (100%).

**Figure 4:**
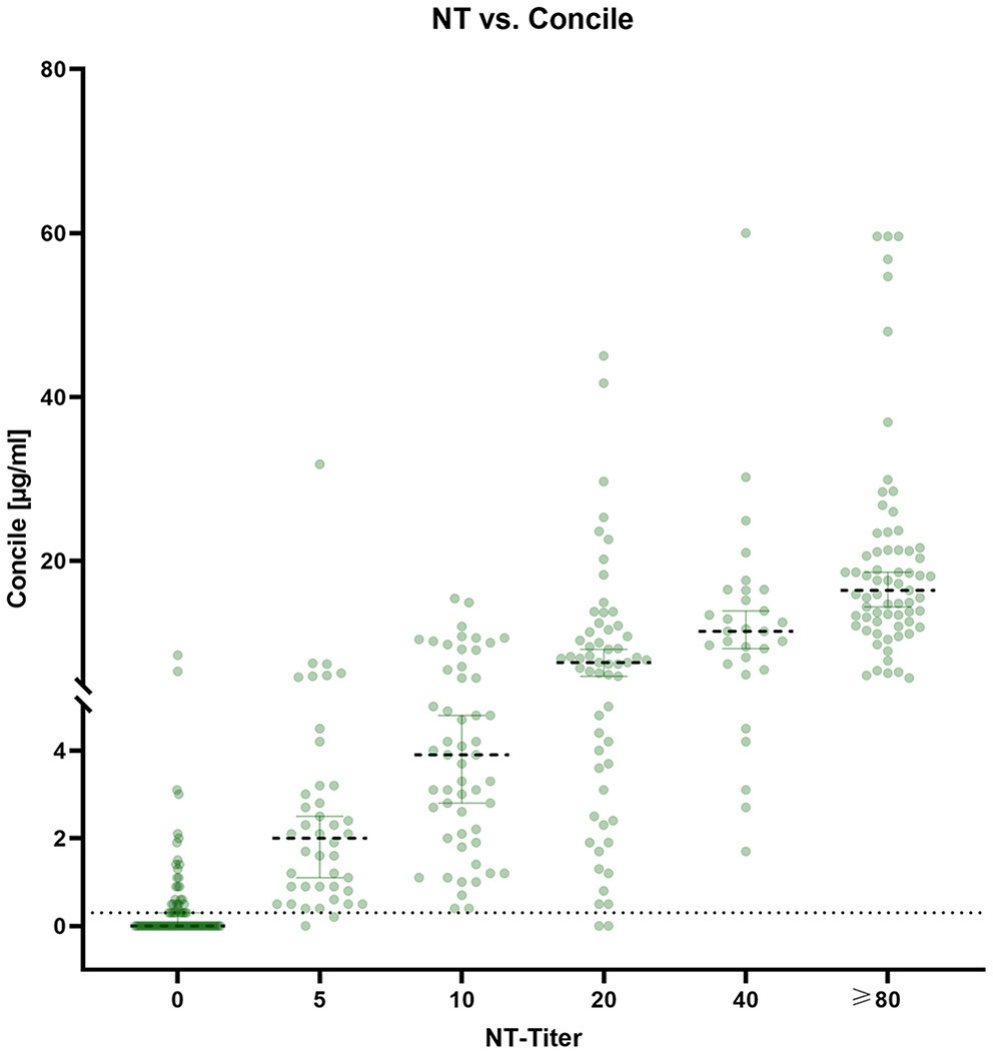
Plot of the percentage of positive results of all four tests in comparison to the respective NT titer.

The percentage of positive results of all four tests in relation to the results obtained by NT is shown in Figure 4. While both the Coris (light green) and the NeutraLISA (light blue) have moderately low levels of false positive test results for NT-negative samples (i.e. 10.7 and 17.8%), the Concile and AdipoGen both have similarly high numbers of false positive among NT-negative samples (i.e. 41.7 and 45.2%). However, when looking at NT-positive samples, the Concile and AdipoGen have consistently good detection rates of ≥ 94% among all titer levels. Detection rates of the NeutraLISA improve with rising NT titers and reach 100% for samples with a titer ≥40 along with the Concile and the AdipoGen. Detection rates of the Coris also gradually improve with rising titers but remain overall low compared to the other three tests (the only exception being samples with a titer of ≥80).

The correlation between all four immunoassays as well as the correlation of each assay with the NT are shown in Table 3. Correlations were mostly medium to strong with the only exception of the Coris NP assay. The closest correlation (0.96) was observed between both sELISAs followed by the NeutraLISA and the Concile (0.9). A medium correlation (0.8–0.85) was demonstrated between all assays and the NT with the NeutraLISA exhibiting the closest correlation to the NT. In general, the Coris exhibited the weakest correlations with all other assays including the weakest correlation (0.67) that was observed between the Coris and the AdipoGen. As expected, no correlations at all were observed between the Coris specifically targeted at NP-specific antibodies and all other (spike protein specific) assays.

**Table 3:** Correlation between all immunoassays and with NT.

	**NT**	**AdipoGen**	**NeutraLISA**	**Coris (NP)**	**Coris**	**Concile**
NT	1,00	0,84	0,85	0,13	0,80	0,84
AdipoGen		1,00	0,96	−0,17	0,67	0,83
NeutraLISA			1,00	−0,12	0,73	0,90
Coris (NP)				1,00	0,19	0,13
Coris					1,00	0,76
Concile						1,00

*All coefficients are significant (p < 0.0001) with the only exception of the Coris NP assay; correlations are marked in color; strong correlations are marked in green shades while weaker correlations are marked in yellow, orange and red*.

As the Coris not only detects RBD specific antibodies, but also antibodies against the nucleocapsid protein (NP), we examined all samples from convalescent patients (*n* = 128) with a previous PCR-confirmed infection specifically for antibodies against NP. NP specific antibodies were detected in a little over half of the samples (83/128) resulting in a NP specific sensitivity of 65%. At the same time, we also tested the 30 negative control samples, of which all were NP negative, giving a specificity of 100%. We did not include the samples from partially/fully vaccinated individuals, as our information is not sufficient to exclude a past SARS-CoV-2 infection with absolute certainty.

### Antibody Quantities Determined by the Concile (InfectCheck COVID-19 Nab)

**Figure 5:**
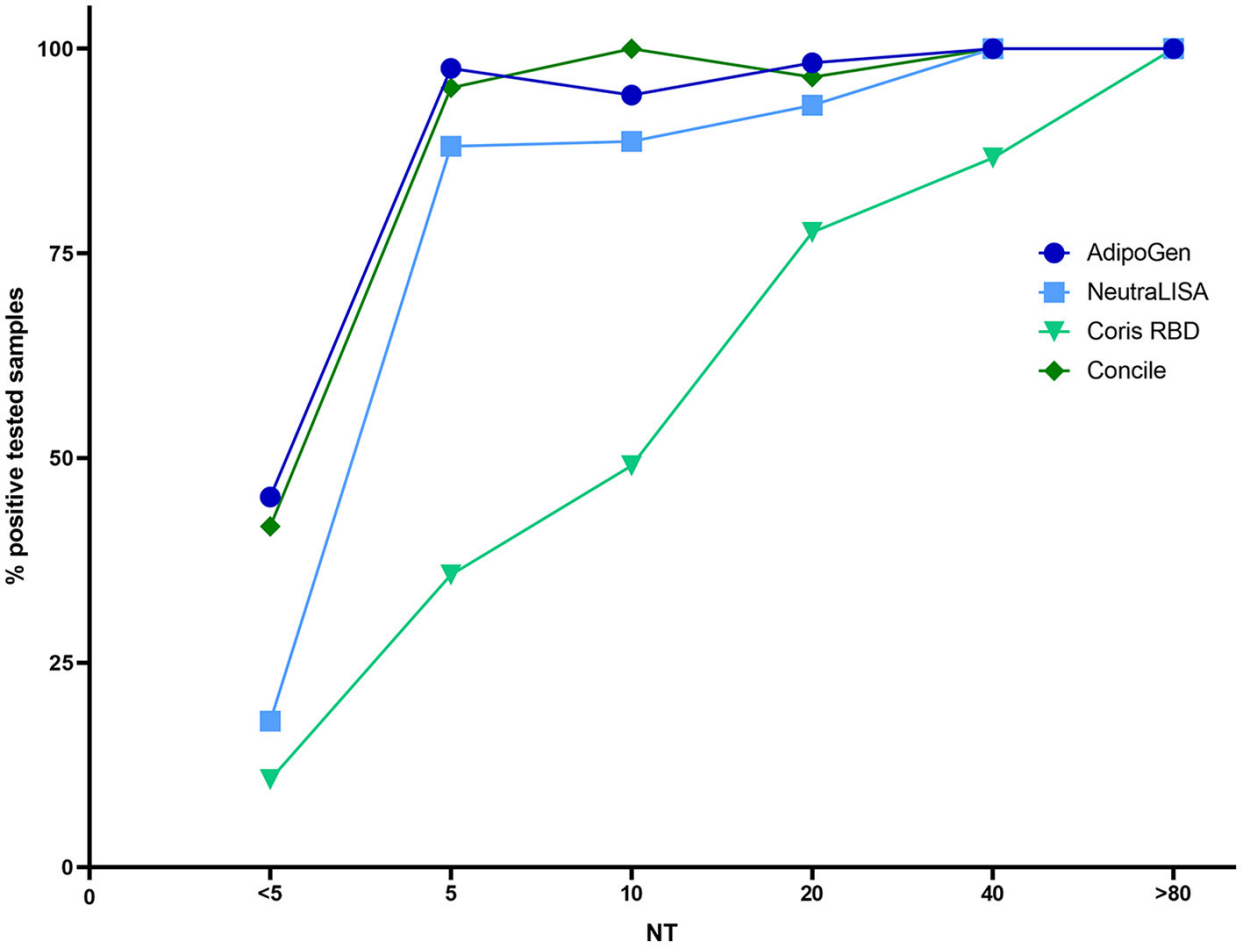
Distribution of antibody quantities (μg/ml) determined by the Concile InfectCheck COVID-19 Nab in relation to the results of the NT. In accordance with the manufacturer's instructions, cut-off was set to 0.3 μg/ml. Despite a wide distribution of results (R^2^ 0.3, *p* < 0.0001), a low-grade linear trend could be observed with the highest precision observed for samples with high NT-titers (≥80).

In addition to qualitative results, the Concile LFA also offers quantification of antibodies (μg/ml). An overview of quantitative results is given in Figure 5. Despite a noticeable wide distribution of results within each NT titer level, a low grade linear trend could still be observed (R^2^ 0.3, *p* < 0.0001). The highest precision was observed for samples with NT titer levels ≥80.

## Discussion

Infection with SARS-CoV-2 elicits a humoral immune response that results in antibodies against specific viral proteins, including neutralizing antibodies (NAb). In this study, we investigated four immunassays, i.e. two surrogate ELISAs (sELISA) and two lateral flow assays (LFA), specifically designed for the detection of SARS-CoV-2 NAbs and evaluated their sensitivity and specificity in direct comparison to a gold standard virus based method (NT).

Of the 334 serum samples evaluated in this study, the majority came from convalescent patients (38%) and fully vaccinated individuals (41%). Interestingly, mean NT titers differed markedly between convalescent and vaccinated individuals, with titers nearly four times higher in the latter. In addition, we were able to detect NAb in all samples from vaccinated individuals while 11% of samples from convalescent patients were negative for NAb. This is also in line with a recent study by Cavanaugh et al. who found that a full vaccination provides significantly better protection against reinfection compared to natural infection (18). Remarkably, no NAb at all could be detected in partially vaccinated individuals. This observation was somewhat surprising because it contrasts with other studies in which at least low levels of neutralizing antibodies were detected after primary vaccination (19, 20). Since the samples were collected at similar time points after vaccination, this discrepancy is likely due to differences in testing methods. Our NT was essentially designed as a PRNT100 to maximize specificity and detect actual protective neutralizing antibodies with the highest possible confidence. However, this approach implies a reduction in test sensitivity and may explain the differences with studies using other methods such as PRNT50 or surrogate ELISA tests. This explanation is also supported by our finding that, although the NT results were negative, both sELISAs and both LFAs tested in this study detected NAb in many samples from partially vaccinated individuals.

The highest overall sensitivity (98.4%) was observed for the Concile despite it being a LFA, closely followed by the AdipoGen sELISA (98%). In contrast, sensitivity was lower for the NeutraLISA sELISA (86%) while the Coris exhibited the least sensitivity (71.6%). However, while the NeutraLISA demonstrated a similar overall specificity of 82.1%, the AdipoGen and Concile both exhibited a markedly lower overall specificity (54.8 and 58.3% respectively). In contrast, the Coris was the only test with a specificity (89.3%) higher than the sensitivity. Sensitivity of all four tests was highest when testing samples of fully vaccinated individuals. The fact that the Coris LFA offers the simultaneous detection of NP specific antibodies is potentially interesting. However, the NP specific sensitivity of only 65% is not sufficient to reliably confirm or exclude a previous infection. The low specificity of the Concile and the AdipoGen is due to a large number of apparent false positive results within the groups of partially vaccinated and convalescent individuals. In this context, we observed strikingly different specificities when testing different subgroups. Most interestingly, all four tests exhibited a specificity of 100% in the group of negative control samples, i.e. samples taken prior to the appearance of SARS-CoV-2. This is in stark contrast to the specificities determined based on testing samples from partially vaccinated individuals, which were as low as 25%. Similar results were observed for NT-negative samples from convalescent individuals, raising the question if these samples are truly negative. As already mentioned above, this discrepancy could be due to the fact that our NT is very conservatively designed to be as specific as possible. This limitation might lead to an underestimation of specificity of the evaluated test. In this context, the observation that samples after prime immunization have detectable but generally low antibody levels (20–22) is consistent. At the same time, another possible explanation for this discrepancy could be the presence of SARS-CoV-2 specific antibodies without neutralizing activity. Such non-neutralizing antibodies may cross-react with both sELISA and LFA and lead to false positive results due to their artificial nature. In addition, we observed only very little correlation (R^2^ 0.26, *p* < 0.0001) between the entirety of IgG antibodies and NAb, including high levels of antibodies but no neutralizing activity which could favor cross-reactivity. The wide distribution of inhibition values that was observed for both sELISAs visualizes the overall low specificity yet high sensitivity. At the same time, the two dimensional distribution shows a wide spread of inhibition values, especially within negative to low NT titers, and underlines the non-quantitative character of both tests. Thus, no conclusion about titer levels can be drawn from inhibition values determined by either sELISA, which is a clear disadvantage compared to NT. The possibility of antibody quantification by the Concile LFA is potentially appealing. However, due to the wide distribution of results within each titer level together with the low overall specificity, the clinical use and interpretation of quantitative results remains questionable.

Another aspect to consider when assessing and interpreting these results is the different conceptual design of the four assays. As described and illustrated in Figure 2, both competitive sELISAs are similar in design and mimic the interaction of RBD and the ACE2 receptor to determine the inhibitory effect of neutralizing antibodies that are capable of interrupting this interaction. Therefore, they can at least artificially test the biological function of RBD-specific NAb. However, NAb are directed against different viral structures (23), which in principle gives virus neutralization assays a clear advantage in terms of biological sensitivity because they can detect all NAb regardless of the target structure. The fact that both sELISAs show high sensitivities despite this methodological disadvantage is likely due to the fact, that the vast majority of NAb was shown to be directed against the RBD (5). Therefore, their imperfect sensitivity is likely due to a combination of the disadvantage in biological sensitivity and the performance of the test itself (i.e. technical sensitivity). While the same study has also shown that not all RBD-specific antibodies are neutralizing, this could still explain the high sensitivity of the Concile LFA. Although it only detects RBD-specific antibodies without assessing functionality. While this should have a detrimental effect on (biological) sensitivity, it is again likely that at least a large proportion of these RBD-specific antibodies are in fact able to neutralize. Admittedly, this is in contrast to the significantly lower sensitivity of the Coris LFA, which also detects RBD-specific antibodies. However, this might be a methodological issue as the Concile uses anti-human-IgG for capture and gold conjugated RBD for detection while the Coris works the other way around and uses RBD for capture and gold conjugated anti-human-IgG for detection, which seems to lead to a lower technical sensitivity and should be explored in more detail in further studies.

Currently, there is a global effort to promote SARS-CoV-2 immunization. However, many countries have recently reported stagnating numbers of vaccinations. At the same time, countries are trying to return to normality and gradually ease restrictions, mostly for fully vaccinated people, while at the same time reports have begun to emerge about suboptimal immune responses after vaccination, especially among risk groups (24–26). Albeit still missing data on correlate of protection (27), this has led to an increased demand for NAbs testing to assess vaccination success. In addition, recent publications have indicated a beneficial effect of booster vaccinations to mitigate the reported decline in NAb (28–31), which has further increased the demand for NAb testing to select eligible groups.

Based on the results of this study, both sELISAs are suitable to be used as alternative testing methods in determining NAb levels post immunization (and with minor compromises also post infection) when NT testing is not available in standard laboratories. It must be noted, however, that all tests seem unsuitable to test patient cohorts with an expected low immune responses (e.g. patients under B cell depletion therapy, oncology patients and overall immunocompromised patients etc.) as there is probably a high risk of false positive results due to the previously described potential cross-reactivity with non-neutralizing antibodies.

A huge advantage of both sELISAs is, of course, the significantly reduces turn-around time of only a few hours compared to several days. The same can be said about the Concile LFA, which can be used by medical practices and other non-laboratory institutions due to its quick and easy workflow and turn-around of only 20 min.

## Data Availability Statement

The original contributions presented in the study are included in the article/Supplementary Material, further inquiries can be directed to the corresponding author.

## Ethics Statement

Ethical review and approval was not required for the study on human participants in accordance with the local legislation and institutional requirements. Written informed consent for participation was not required for this study in accordance with the national legislation and the institutional requirements.

## Author Contributions

PG and KZ: conceptualization, methodology, visualization, writing – review and editing. HV and RW: data curation, writing – review and editing. KM: conceptualization, methodology, writing – original draft, and visualization. All authors contributed to the article and approved the submitted version.

## Funding

This study was funded by the Medical Biodefense Research Program of the Bundeswehr Medical Service.

## Conflict of Interest

The authors declare that the research was conducted in the absence of any commercial or financial relationships that could be construed as a potential conflict of interest.

## Publisher's Note

All claims expressed in this article are solely those of the authors and do not necessarily represent those of their affiliated organizations, or those of the publisher, the editors and the reviewers. Any product that may be evaluated in this article, or claim that may be made by its manufacturer, is not guaranteed or endorsed by the publisher.
