# Supplementary material for: Evaluation of Two Rapid Lateral Flow Tests and Two Surrogate ELISAs for the Detection of SARS-CoV-2 Specific Neutralizing Antibodies
Source: Front Med (Lausanne). 2022 Feb 4;9:820151. doi: 10.3389/fmed.2022.820151 (PMC8854747; doi:10.3389/fmed.2022.820151)
Supplement: Supplementary file 1 [file Data_Sheet_1.docx]

Supplementary Material

**Supplementary Table 1. Commercial SARS-CoV-2 IgG ELISA and NT results results of all samples tested in this study.** Groups: C=Convalescent, V=Fully vaccinated, PV=Partially vaccinated, NC=Negative control samples

| **Stud. ID** | **Euroimmun IgG Result/Ratio** | **NT titer** | **Group** |
| --- | --- | --- | --- |
| ID_001 | 0,44 | 0 | **C** |
| ID_002 | 1,10 | 0 | **C** |
| ID_003 | 13,36 | 0 | **C** |
| ID_004 | 2,18 | 0 | **C** |
| ID_005 | 3,85 | 0 | **C** |
| ID_006 | 0,44 | 0 | **C** |
| ID_007 | 0,44 | 0 | **C** |
| ID_008 | 1,01 | 0 | **C** |
| ID_009 | 0,83 | 0 | **C** |
| ID_010 | 6,74 | 0 | **C** |
| ID_011 | 2,33 | 0 | **C** |
| ID_012 | 6,34 | 20 | **C** |
| ID_013 | 1,25 | 10 | **C** |
| ID_014 | 0,95 | 0 | **C** |
| ID_015 | 0,56 | 5 | **C** |
| ID_016 | 1,13 | 5 | **C** |
| ID_017 | 1,90 | 0 | **C** |
| ID_018 | 6,11 | 5 | **C** |
| ID_019 | 5,31 | 20 | **C** |
| ID_020 | 9,04 | 320 | **C** |
| ID_021 | 0,20 | 5 | **C** |
| ID_022 | 7,68 | 20 | **C** |
| ID_023 | 0,89 | 0 | **C** |
| ID_024 | 0,31 | 20 | **C** |
| ID_025 | 0,36 | 20 | **C** |
| ID_026 | 1,53 | 10 | **C** |
| ID_027 | 3,56 | 20 | **C** |
| ID_028 | 1,82 | 20 | **C** |
| ID_029 | 0,33 | 20 | **C** |
| ID_030 | 5,06 | 10 | **C** |
| ID_031 | 2,21 | 20 | **C** |
| ID_032 | 6,64 | 5 | **C** |
| ID_033 | 5,88 | 10 | **C** |
| ID_034 | 1,02 | 10 | **C** |
| ID_035 | 6,97 | 40 | **C** |
| ID_036 | 5,94 | 40 | **C** |
| ID_037 | 0,98 | 20 | **C** |
| ID_038 | 5,67 | 80 | **C** |
| ID_039 | 8,92 | 80 | **C** |
| ID_040 | 1,35 | 10 | **C** |
| ID_041 | 4,37 | 20 | **C** |
| ID_042 | 8,33 | 80 | **C** |
| ID_043 | 9,12 | 20 | **C** |
| ID_044 | 4,70 | 40 | **C** |
| ID_045 | 3,12 | 40 | **C** |
| ID_046 | 2,51 | 40 | **C** |
| ID_047 | 10,16 | 160 | **C** |
| ID_048 | 10,21 | 640 | **C** |
| ID_049 | 5,85 | 80 | **C** |
| ID_050 | 5,83 | 80 | **C** |
| ID_051 | 2,00 | 5 | **C** |
| ID_052 | 3,77 | 5 | **C** |
| ID_053 | 7,27 | 40 | **C** |
| ID_054 | 1,73 | 5 | **C** |
| ID_055 | 9,15 | 640 | **C** |
| ID_056 | 10,09 | 160 | **C** |
| ID_057 | 1,90 | 5 | **C** |
| ID_058 | 4,49 | 40 | **C** |
| ID_059 | 1,89 | 5 | **C** |
| ID_060 | 1,94 | 5 | **C** |
| ID_061 | 4,13 | 80 | **C** |
| ID_062 | 4,55 | 10 | **C** |
| ID_063 | 1,53 | 5 | **C** |
| ID_064 | 2,67 | 10 | **C** |
| ID_065 | 5,79 | 20 | **C** |
| ID_066 | 5,25 | 40 | **C** |
| ID_067 | 2,00 | 5 | **C** |
| ID_068 | 3,65 | 10 | **C** |
| ID_069 | 3,75 | 20 | **C** |
| ID_070 | 7,62 | 20 | **C** |
| ID_071 | 1,98 | 5 | **C** |
| ID_072 | 4,10 | 5 | **C** |
| ID_073 | 7,16 | 20 | **C** |
| ID_074 | 4,01 | 10 | **C** |
| ID_075 | 2,93 | 10 | **C** |
| ID_076 | 6,89 | 20 | **C** |
| ID_077 | 6,98 | 40 | **C** |
| ID_078 | 7,78 | 80 | **C** |
| ID_079 | 1,93 | 5 | **C** |
| ID_080 | 4,16 | 5 | **C** |
| ID_081 | 3,46 | 5 | **C** |
| ID_082 | 3,84 | 20 | **C** |
| ID_083 | 2,29 | 5 | **C** |
| ID_084 | 8,08 | 40 | **C** |
| ID_085 | 1,90 | 10 | **C** |
| ID_086 | 4,79 | 20 | **C** |
| ID_087 | 4,01 | 10 | **C** |
| ID_088 | 4,76 | 10 | **C** |
| ID_089 | 5,79 | 20 | **C** |
| ID_090 | 4,81 | 5 | **C** |
| ID_091 | 4,42 | 10 | **C** |
| ID_092 | 4,19 | 5 | **C** |
| ID_093 | 3,80 | 5 | **C** |
| ID_094 | 5,25 | 10 | **C** |
| ID_095 | 5,66 | 20 | **C** |
| ID_096 | 4,73 | 5 | **C** |
| ID_097 | 7,04 | 20 | **C** |
| ID_098 | 5,43 | 10 | **C** |
| ID_099 | 2,41 | 5 | **C** |
| ID_100 | 4,70 | 20 | **C** |
| ID_101 | 4,21 | 10 | **C** |
| ID_102 | 3,28 | 10 | **C** |
| ID_103 | 8,40 | 80 | **C** |
| ID_104 | 15,84 | 80 | **C** |
| ID_105 | 5,10 | 20 | **C** |
| ID_106 | 1,73 | 10 | **C** |
| ID_107 | 4,53 | 10 | **C** |
| ID_108 | 4,14 | 5 | **C** |
| ID_109 | 1,43 | 5 | **C** |
| ID_110 | 3,45 | 5 | **C** |
| ID_111 | 3,83 | 10 | **C** |
| ID_112 | 6,13 | 20 | **C** |
| ID_113 | 1,68 | 5 | **C** |
| ID_114 | 2,09 | 10 | **C** |
| ID_115 | 9,04 | 40 | **C** |
| ID_116 | 34,99 | 320 | **C** |
| ID_117 | 2,10 | 5 | **C** |
| ID_118 | 4,67 | 20 | **C** |
| ID_119 | 9,11 | 20 | **C** |
| ID_120 | 2,02 | 5 | **C** |
| ID_121 | 10,64 | 5 | **C** |
| ID_122 | 5,78 | 20 | **C** |
| ID_123 | 4,65 | 20 | **C** |
| ID_124 | 3,87 | 10 | **C** |
| ID_125 | 1,19 | 5 | **C** |
| ID_126 | 5,49 | 20 | **C** |
| ID_127 | 1,11 | 5 | **C** |
| ID_128 | > 36 | 320 | **C** |
| ID_129 | positive | 10 | **V** |
| ID_130 | positive | 20 | **V** |
| ID_131 | positive | 40 | **V** |
| ID_132 | positive | 20 | **V** |
| ID_133 | positive | 40 | **V** |
| ID_134 | positive | 10 | **V** |
| ID_135 | positive | 20 | **V** |
| ID_136 | positive | 5 | **V** |
| ID_137 | positive | 5 | **V** |
| ID_138 | positive | 80 | **V** |
| ID_139 | positive | 10 | **V** |
| ID_140 | positive | 40 | **V** |
| ID_141 | positive | 20 | **V** |
| ID_142 | positive | 10 | **V** |
| ID_143 | positive | 10 | **V** |
| ID_144 | positive | 10 | **V** |
| ID_145 | positive | 10 | **V** |
| ID_146 | positive | 20 | **V** |
| ID_147 | positive | 5 | **V** |
| ID_148 | positive | 20 | **V** |
| ID_149 | positive | 20 | **V** |
| ID_150 | positive | 5 | **V** |
| ID_151 | positive | 20 | **V** |
| ID_152 | positive | 20 | **V** |
| ID_153 | positive | 10 | **V** |
| ID_154 | positive | 10 | **V** |
| ID_155 | positive | 10 | **V** |
| ID_156 | positive | 20 | **V** |
| ID_157 | positive | 20 | **V** |
| ID_158 | positive | 5 | **V** |
| ID_159 | positive | 5 | **V** |
| ID_160 | positive | 10 | **V** |
| ID_161 | positive | 5 | **V** |
| ID_162 | positive | 10 | **V** |
| ID_163 | positive | 10 | **V** |
| ID_164 | positive | 10 | **V** |
| ID_165 | positive | 10 | **V** |
| ID_166 | positive | 20 | **V** |
| ID_167 | positive | 20 | **V** |
| ID_168 | positive | 10 | **V** |
| ID_169 | positive | 20 | **V** |
| ID_170 | positive | 20 | **V** |
| ID_171 | positive | 10 | **V** |
| ID_172 | positive | 40 | **V** |
| ID_173 | positive | 20 | **V** |
| ID_174 | positive | 20 | **V** |
| ID_175 | positive | 10 | **V** |
| ID_176 | positive | 40 | **V** |
| ID_177 | positive | 20 | **V** |
| ID_178 | positive | 10 | **V** |
| ID_179 | positive | 40 | **V** |
| ID_180 | positive | 10 | **V** |
| ID_181 | positive | 40 | **V** |
| ID_182 | positive | 20 | **V** |
| ID_183 | positive | 80 | **V** |
| ID_184 | positive | 20 | **V** |
| ID_185 | positive | 5 | **V** |
| ID_186 | positive | 20 | **V** |
| ID_187 | positive | 20 | **V** |
| ID_188 | positive | 20 | **V** |
| ID_189 | positive | 20 | **V** |
| ID_190 | positive | 5 | **V** |
| ID_191 | positive | 10 | **V** |
| ID_192 | positive | 10 | **V** |
| ID_193 | positive | 20 | **V** |
| ID_194 | positive | 40 | **V** |
| ID_195 | positive | 10 | **V** |
| ID_196 | positive | 10 | **V** |
| ID_197 | positive | 20 | **V** |
| ID_198 | positive | 40 | **V** |
| ID_199 | positive | 40 | **V** |
| ID_200 | positive | 40 | **V** |
| ID_201 | positive | 80 | **V** |
| ID_202 | positive | 20 | **V** |
| ID_203 | positive | 40 | **V** |
| ID_204 | positive | 20 | **V** |
| ID_205 | positive | 10 | **V** |
| ID_206 | positive | 10 | **V** |
| ID_207 | positive | 10 | **V** |
| ID_208 | positive | 10 | **V** |
| ID_209 | 8,51 | 80 | **V** |
| ID_210 | 8,87 | 320 | **V** |
| ID_211 | 8,79 | 640 | **V** |
| ID_212 | 8,44 | 320 | **V** |
| ID_213 | 8,11 | 80 | **V** |
| ID_214 | 9,19 | 640 | **V** |
| ID_215 | 9,11 | 320 | **V** |
| ID_216 | 8,33 | 160 | **V** |
| ID_217 | 8,21 | 160 | **V** |
| ID_218 | 8,51 | 320 | **V** |
| ID_219 | 8,74 | 320 | **V** |
| ID_220 | 8,46 | 160 | **V** |
| ID_221 | 9,11 | 320 | **V** |
| ID_222 | 8,45 | 320 | **V** |
| ID_223 | 8,41 | 40 | **V** |
| ID_224 | 8,22 | 320 | **V** |
| ID_225 | 8,59 | 160 | **V** |
| ID_226 | 9,06 | 640 | **V** |
| ID_227 | 8,59 | 40 | **V** |
| ID_228 | 8,71 | 160 | **V** |
| ID_229 | 8,19 | 40 | **V** |
| ID_230 | 6,10 | 80 | **V** |
| ID_231 | 8,01 | 160 | **V** |
| ID_232 | 6,07 | 160 | **V** |
| ID_233 | 8,53 | 40 | **V** |
| ID_234 | 8,31 | 80 | **V** |
| ID_235 | 9,03 | 640 | **V** |
| ID_236 | 7,55 | 80 | **V** |
| ID_237 | 8,94 | 640 | **V** |
| ID_238 | 8,35 | 160 | **V** |
| ID_239 | 7,77 | 320 | **V** |
| ID_240 | 6,52 | 40 | **V** |
| ID_241 | 8,37 | 320 | **V** |
| ID_242 | 7,63 | 80 | **V** |
| ID_243 | 7,49 | 160 | **V** |
| ID_244 | 8,27 | 640 | **V** |
| ID_245 | 7,88 | 640 | **V** |
| ID_246 | 8,27 | 640 | **V** |
| ID_247 | 7,55 | 320 | **V** |
| ID_248 | 6,92 | 40 | **V** |
| ID_249 | 8,94 | 640 | **V** |
| ID_250 | 8,40 | 320 | **V** |
| ID_251 | 8,64 | 320 | **V** |
| ID_252 | 9,14 | 640 | **V** |
| ID_253 | 3,49 | 10 | **V** |
| ID_254 | 7,70 | 80 | **V** |
| ID_255 | 8,52 | 640 | **V** |
| ID_256 | 7,62 | 320 | **V** |
| ID_257 | 1,70 | 80 | **V** |
| ID_258 | 7,31 | 160 | **V** |
| ID_259 | 7,72 | 640 | **V** |
| ID_260 | 8,06 | 320 | **V** |
| ID_261 | 8,40 | 640 | **V** |
| ID_262 | 7,80 | 160 | **V** |
| ID_263 | 7,67 | 160 | **V** |
| ID_264 | 6,20 | 40 | **V** |
| ID_265 | 3,15 | 0 | **PV** |
| ID_266 | 0,21 | 0 | **PV** |
| ID_267 | 2,45 | 0 | **PV** |
| ID_268 | 5,08 | 0 | **PV** |
| ID_269 | 1,30 | 0 | **PV** |
| ID_270 | 4,99 | 0 | **PV** |
| ID_271 | 2,28 | 0 | **PV** |
| ID_272 | 0,33 | 0 | **PV** |
| ID_273 | 2,16 | 0 | **PV** |
| ID_274 | 2,97 | 0 | **PV** |
| ID_275 | 4,80 | 0 | **PV** |
| ID_276 | 0,09 | 0 | **PV** |
| ID_277 | 6,10 | 0 | **PV** |
| ID_278 | 1,58 | 0 | **PV** |
| ID_279 | 0,80 | 0 | **PV** |
| ID_280 | 3,35 | 0 | **PV** |
| ID_281 | 2,92 | 0 | **PV** |
| ID_282 | 6,26 | 0 | **PV** |
| ID_283 | 4,31 | 0 | **PV** |
| ID_284 | 4,78 | 0 | **PV** |
| ID_285 | 0,66 | 0 | **PV** |
| ID_286 | 6,60 | 0 | **PV** |
| ID_287 | 3,19 | 0 | **PV** |
| ID_288 | 6,38 | 0 | **PV** |
| ID_289 | 1,12 | 0 | **PV** |
| ID_290 | 3,34 | 0 | **PV** |
| ID_291 | 1,55 | 0 | **PV** |
| ID_292 | 1,26 | 0 | **PV** |
| ID_293 | 3,67 | 0 | **PV** |
| ID_294 | 3,20 | 0 | **PV** |
| ID_295 | 1,77 | 0 | **PV** |
| ID_296 | 5,60 | 0 | **PV** |
| ID_297 | 4,25 | 0 | **PV** |
| ID_298 | 2,69 | 0 | **PV** |
| ID_299 | 2,49 | 0 | **PV** |
| ID_300 | 1,26 | 0 | **PV** |
| ID_301 | 1,25 | 0 | **PV** |
| ID_302 | 2,30 | 0 | **PV** |
| ID_303 | 2,43 | 0 | **PV** |
| ID_304 | 2,72 | 0 | **PV** |
| ID_305 | 0,20 | 0 | **NC** |
| ID_306 | 0,24 | 0 | **NC** |
| ID_307 | 0,05 | 0 | **NC** |
| ID_308 | 0,14 | 0 | **NC** |
| ID_309 | 0,08 | 0 | **NC** |
| ID_310 | 0,13 | 0 | **NC** |
| ID_311 | 0,31 | 0 | **NC** |
| ID_312 | 0,17 | 0 | **NC** |
| ID_313 | 0,25 | 0 | **NC** |
| ID_314 | 0,15 | 0 | **NC** |
| ID_315 | 0,09 | 0 | **NC** |
| ID_316 | 0,20 | 0 | **NC** |
| ID_317 | 0,57 | 0 | **NC** |
| ID_318 | 0,62 | 0 | **NC** |
| ID_319 | 0,21 | 0 | **NC** |
| ID_320 | 0,26 | 0 | **NC** |
| ID_321 | 0,08 | 0 | **NC** |
| ID_322 | 0,11 | 0 | **NC** |
| ID_323 | 0,17 | 0 | **NC** |
| ID_324 | 0,24 | 0 | **NC** |
| ID_325 | 0,31 | 0 | **NC** |
| ID_326 | 0,15 | 0 | **NC** |
| ID_327 | 0,17 | 0 | **NC** |
| ID_328 | 0,12 | 0 | **NC** |
| ID_329 | 0,20 | 0 | **NC** |
| ID_330 | 0,12 | 0 | **NC** |
| ID_331 | 0,08 | 0 | **NC** |
| ID_332 | 0,14 | 0 | **NC** |
| ID_333 | 0,08 | 0 | **NC** |
| ID_334 | 0,16 | 0 | **NC** |

**Supplementary Figure 1.** In-house visual analog scale for standardized assessment of line intensity of colloidal gold lateral flow assays.

**Supplementary Figure 2. (A) Distribution of inhibition values determined by AdipoGen (left) and NeutraLISA (right)** The wide distribution of inhibition values visualizes the overall low specificity yet higher sensitivity of both sELISA  **(B) Two dimensional distribution of inhibition values among titer levels** The wide spread of inhibition values, especially within negative to low NT iters, underlines the non-quantitative character of both tests and diesn not allow any conclusions on titer levels.

**A**


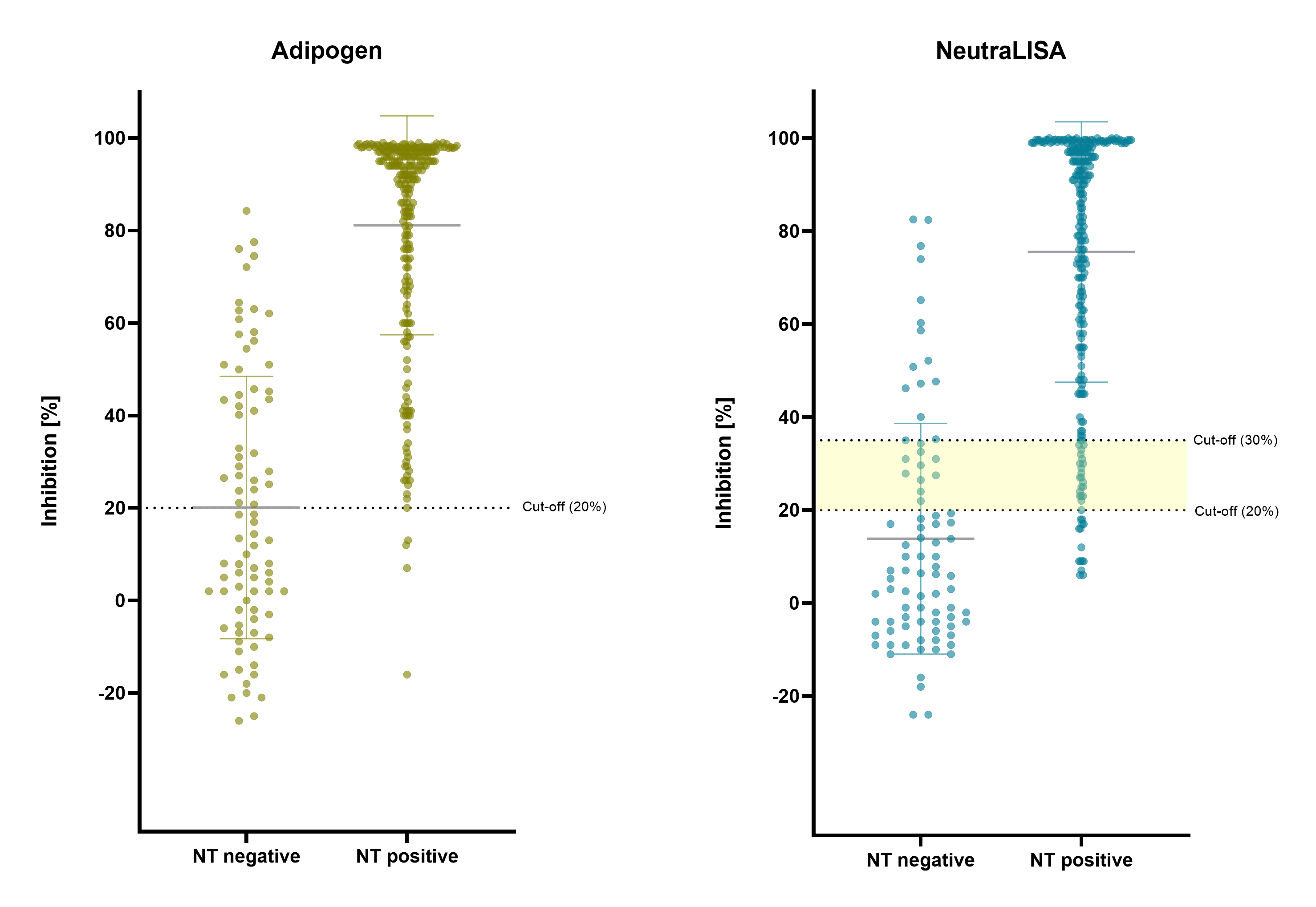


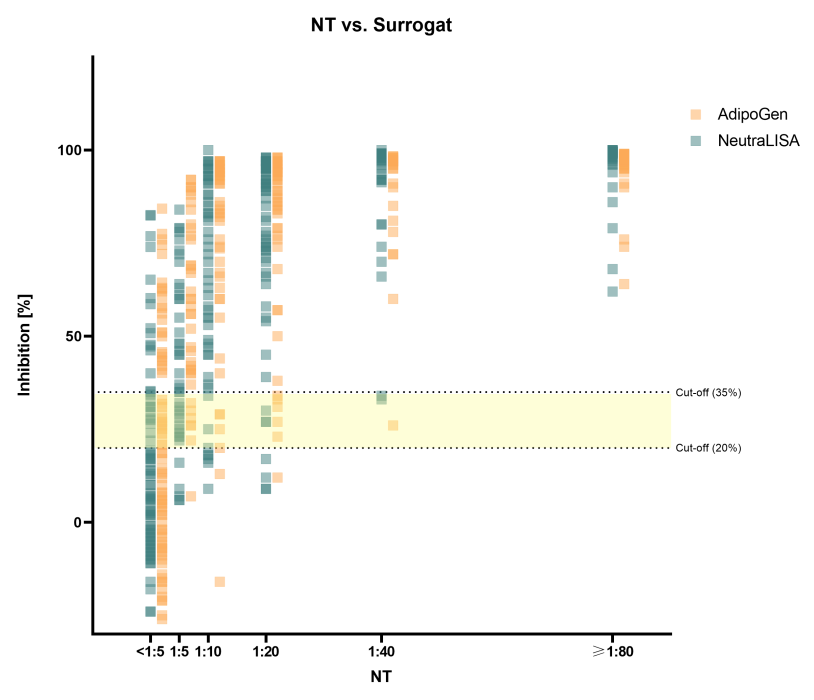


**B**
